# Anaphylaxis events in the PIONEER study of avapritinib in indolent systemic mastocytosis^☆^^☆☆^

**DOI:** 10.1016/j.waojou.2026.101352

**Published:** 2026-03-14

**Authors:** Thanai Pongdee, Mariana Castells, Cem Akin, Ingunn Dybedal, Jason Gotlib, Jens Peter Panse, Ivan Alvarez-Twose, Cristina Morales Cabeza, Sonia Cerquozzi, Peter Vadas, Vidushi Swarup, Pankit Vachhani, Friederike Wortmann, Cecilia Arana Yi, Ilda Bidollari, Kate Newberry, Daniel Shaheen, Karin Hartmann

**Affiliations:** a Division of Allergic Diseases, Mayo Clinic, Rochester, MN, USA; b Division of Allergy and Clinical Immunology, Department of Medicine, Brigham and Women's Hospital, Harvard Medical School, Hale Building for Transformative Medicine, Boston, MA, USA; c Division of Allergy and Clinical Immunology, University of Michigan, Ann Arbor, MI, USA; d Department of Hematology, Oslo University Hospital, Rikshospitalet, Oslo, Norway; e Clinical Research Unit, Pharmacological Department, Oslo University Hospital, Oslo, Norway; f Division of Hematology, Stanford Cancer Institute Stanford University School of Medicine, Stanford, CA, USA; g Department of Oncology, Hematology, Hemostaseology and Stem Cell Transplantation, University Hospital RWTH Aachen, Aachen, Germany; h Center for Integrated Oncology (CIO), Aachen Bonn Cologne Düsseldorf (ABCD), Aachen, Germany; i Instituto de Estudios de Mastocitosis de Castilla-La Mancha (CLMast), Reference Center for Mastocytosis and CIBERONC, Toledo, Spain; j Spanish Network on Mastocytosis (REMA), Toledo and Salamanca, Spain; k Instituto de Investigación Sanitaria de Castilla-La Mancha (IDISCAM), Toledo, Spain; l Instituto de Estudios de Mastocitosis de Castilla La Mancha, Hospital Virgen del Valle, Toledo, Spain; m Division of Hematology and Hematologic Malignancies, University of Calgary, Calgary, Alberta, Canada; n Division of Clinical Immunology and Allergy, Department of Medicine, St. Michael's Hospital, Toronto, Ontario, Canada; o St. Michael's Hospital, Toronto, Ontario, Canada; p Division of Hematology and Oncology and O'Neal Comprehensive Cancer Center, Heersink School of Medicine, University of Alabama at Birmingham (UAB), Birmingham, AL, USA; q Klinik für Hämatologie und Onkologie, Lübeck, Germany; r Division of Hematology, Mayo Clinic, Scottsdale, AZ, USA; s Blueprint Medicines Corporation, Cambridge, MA, USA; t Division of Allergy, Department of Dermatology, University Hospital Basel and University of Basel, Basel, Switzerland; u Department of Clinical Research, University Hospital Basel and University of Basel, Basel, Switzerland; v Department of Biomedicine, University Hospital Basel and University of Basel, Basel, Switzerland

**Keywords:** Anaphylaxis, Indolent systemic mastocytosis, Avapritinib

## Abstract

**Background:**

Patients with indolent systemic mastocytosis (ISM), a clonal mast cell disease primarily driven by the KIT D816V mutation, often have lifelong debilitating symptoms. Anaphylaxis is a common feature of the disease seen in up to half of patients. The effects of KIT D816V-targeted therapy on the incidence of anaphylaxis are unknown.

**Methods:**

We describe anaphylaxis events occurring in the study population of PIONEER (NCT03731260) during the 12-week screening and/or 24-week treatment period. This study had previously demonstrated the efficacy and safety of the oral, highly selective, KIT D816V inhibitor avapritinib compared with placebo in patients with moderate-to-severe ISM.

**Results:**

In total, 13/212 (6.1%) patients in PIONEER experienced anaphylaxis during screening or treatment (6 during screening, 5 during treatment, and 2 during both screening and treatment). Baseline demographics, clinical characteristics, and known triggers varied. During the randomized, placebo-controlled treatment period, 4/141 (2.8%) avapritinib-treated patients and 3/71 (4.2%) placebo-treated patients experienced anaphylaxis.

**Conclusions:**

Larger studies with longer-term follow-up are required to further confirm the effects of avapritinib on anaphylaxis in patients with SM.

## Introduction

Systemic mastocytosis (SM) is a clonal mast cell (MC) disease driven by the KIT D816V mutation in >95% of cases.^1^ Patients can endure debilitating symptoms, including life-threatening anaphylaxis. Anaphylaxis frequency varies from 20% to 49% in patients with SM, with particularly frequent occurrence in patients with indolent SM (ISM).^1^^,^^2^ Current management of anaphylaxis in SM includes trigger avoidance, epinephrine, allergen-specific immunotherapy, H1 antihistamines, and omalizumab; however, effective SM-targeted therapies are needed.^1^, ^2^, ^3^ Avapritinib is a selective KIT D816V inhibitor approved for advanced SM and ISM in adults in the United States and Europe.^3^, ^4^, ^5^ The global, randomized, placebo-controlled PIONEER trial had evaluated avapritinib plus best supportive care (BSC) versus placebo plus BSC in patients with moderate-to-severe ISM over 24 weeks.^6^ Overall, avapritinib-treated patients demonstrated significant improvements in symptoms, MC burden, and quality of life (QoL) compared with placebo at 24 weeks of treatment. A total of 24 patients randomized to receive avapritinib and 10 patients randomized to receive placebo had a medical history of anaphylaxis. In the present subanalysis of PIONEER, we report a descriptive analysis of 13 patients with ISM randomized to avapritinib or placebo who experienced anaphylaxis during screening or treatment.

## Patients and methods

The analyses reported in this manuscript are based on an exploratory endpoint and post hoc analysis of data from the PIONEER study. Trial methods for PIONEER were described previously.^6^ Patients with moderate-to-severe ISM symptoms (total symptom score [TSS] ≥28 at screening) despite BSC with ≥2 antimediator drugs were randomized 2:1 to avapritinib 25 mg orally once daily plus BSC (avapritinib) or placebo plus BSC (placebo) for 24 weeks. The primary endpoint was mean change in TSS from baseline to Week 24. The ISM symptom assessment form (ISM-SAF; ©2018 Blueprint Medicines Corporation) was used to assess severity of 11 ISM symptoms. QoL was assessed using the 12-Item Short-Form Health Survey (SF-12) and Mastocytosis Quality of Life Questionnaire (MC-QoL).

Analysis of anaphylactic reactions was a prespecified exploratory endpoint. For analyses detailed here, anaphylaxis events observed during the 12-week screening period and/or the 24-week treatment period were evaluated. Events were included if documented by the treating physician in dedicated case report forms for anaphylaxis events treated with epinephrine and/or documented as an adverse event. For each event, date of known triggers, associated symptoms, and administered treatment were collected. Physicians also completed questionnaires on patient history of anaphylaxis events, known triggers, and changes in patient QoL during treatment. Data were summarized by treatment group using descriptive statistics. A formal statistical comparison between treatment groups was not performed due to the low number of anaphylaxis events during the study period.

## Results

### Demographics, clinical characteristics, and treatment history

In total, 13/212 (6.1%) patients in PIONEER experienced anaphylaxis during screening or treatment (avapritinib, n = 10/141 [7.1%]; placebo, n = 3/71 [4.2%]; Supplemental Table 1). Eleven (84.6%) patients were female, 2 (15.4%) were male. Numbers of known allergens/triggers for anaphylaxis events ranged from 0 to 13. Triggers included foods, medications, Hymenoptera venom, stress, and cold/heat. Four patients (avapritinib, n = 2; placebo, n = 2) experienced at least 1 event for which the trigger was unknown. Patients had received multiple prior treatments for ISM. Five of 13 patients (avapritinib, n = 2; placebo, n = 3) received omalizumab prior to randomization and throughout the treatment period (Supplemental Table 1). One patient received a single dose of omalizumab before enrollment and developed acute allergic symptoms. This patient discontinued omalizumab prior to receiving avapritinib.

### Baseline diagnostics and symptomatology

ISM symptoms of the 13 patients included, in addition to anaphylaxis, cutaneous symptoms (flushing, itching, rashes), gastrointestinal symptoms (abdominal pain, diarrhea, nausea), fatigue, headache, joint/bone pain, and neurological symptoms. Baseline values for TSS ranged between 31.0 and 85.6, basal serum tryptase levels ranged from 3.6 ng/mL to 235.2 ng/mL, and bone marrow MC burden varied from 1% to 25%. Eleven of 13 patients had a detectable *KIT* D816V mutation with an allele burden that ranged from 0.07% to 6.75% (Table 1). Skin involvement by mastocytosis was documented in 10 patients (avapritinib, n = 7; placebo, n = 3).

**Table 1:** Baseline diagnostics and symptomatology.

Patients with anaphylaxis events	Serum tryptase (ng/mL)	Bone marrow mast cells (%)	*KIT* D816V mutation (VAF) using ddPCR^a^ (%)	Skin mast cells (counts/mm^2^), lesional/non-lesional	TSS^b^	GI domain score^c^	Skin domain score^d^	Neuro-cognitive domain score^e^	Skin involvement
**Avapritinib, n = 10/141 during screening and interventional period**^f^									
1	3.6	3	Mutation not detected	150/148	33.0	3.4	11.1	9.7	Yes
2	104.0	20	4.54	587/307	34.8	5.5	12.4	11.5	Yes
3	38.6	15	0.96	NA	57.4	16.9	23.5	18.3	No
4	63.8	7	0.08	237/152	60.6	11.9	18.1	16.1	Yes
5	62.2	20	1.14	967/102	31.3	9.1	10.2	6.2	Yes
6	75.4	25	0.91	376/250	36.5	3.4	5.1	15.4	Yes
7	10.6	1	0.07	NA	74.2	18.9	16.2	22.5	No
8	4.2	3	0.77	NA	34.6	10.2	1.9	7.3	No
9	27.5	5	0.26	385/104	82.5	15.6	21.1	26.5	Yes
10	25.1	7	Mutation not detected	109/117	85.0	18.6	22.5	26.0	Yes
**Placebo, n = 3/71 during screening and interventional period**^g^									
11	25.5	10	0.07	370/148	85.6	21.5	24.5	23.4	Yes
12	235.2	15	6.75	739/311	31.0	0.9	13.1	8.7	Yes
13	26.3	7	0.68	535/72	38.2	4.9	12.5	10.3	Yes

ddPCR, droplet digital polymerase chain reaction; GI, gastrointestinal; ISM = indolent systemic mastocytosis; NA, not available; TSS, total symptom score; VAF, variant allele fraction.

a Limit of detection = 0.02%.

b TSS (range 0–110) is based on severity of 11 ISM symptoms, each assessed from 0 (no symptom) to 10 (worst imaginable symptom).

c GI domain score includes abdominal pain, diarrhea, and nausea.

d Skin domain score includes spots, itching, and flushing.

e Neurocognitive domain score includes brain fog, headache, and dizziness.

f In the avapritinib group, 4/141 (2.8%) patients experienced anaphylaxis events during treatment.

g In the placebo group, 3/71 (4.2%) patients experienced anaphylaxis events while on placebo.

### Anaphylaxis events

**Fig. 1:**
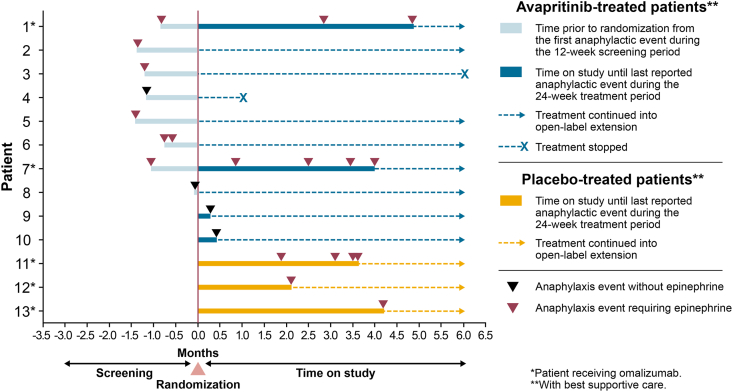
Anaphylaxis events relative to randomization

Median length of follow-up during the randomized, placebo-controlled portion of the study for the 13 patients who experienced anaphylaxis was 5.6 months (range: 0.9–5.6). Four of the 13 (30.7%) patients experienced multiple anaphylaxis events during screening and/or treatment (Fig. 1). In the avapritinib group, 10 patients experienced anaphylaxis: 6 during screening only, 2 during treatment only, and 2 during both screening and treatment. In the placebo group, no patient had anaphylaxis during screening; all 3 patients experienced anaphylaxis during treatment. Restricting the analysis to the interventional period only, anaphylaxis occurred in 4/141 (2.8%) avapritinib-treated patients and 3/71 (4.2%) placebo-treated patients.

### QoL

The patients QoL experience at baseline ranged from being able to perform daily activities to recurrent Emergency Department visits that could negatively impact QoL. Six of the 10 (60%) avapritinib-treated patients had improvement in QoL per investigator assessment: 1 patient (10%) saw worsening, and 3 patients (30%) were without significant change during treatment. Of the 3 placebo-treated patients, 2 (66.6%) had mild improvement, and 1 (33.3%) had no improvement in QoL. QoL improvements during/after avapritinib treatment ranged from reduced medication usage and hospital visits to increased physical functioning and participation in activities. In both treatment groups, numeric improvements in MC-QoL and SF-12 scores were observed; patient numbers were too small to show a statistical significance.

## Discussion

We report on 13 patients in the PIONEER trial who experienced anaphylaxis. Baseline demographics, ISM disease burden, and anaphylaxis triggers varied widely. During the treatment period, 4/141 (2.8%) avapritinib-treated patients and 3/71 (4.2%) placebo-treated patients experienced anaphylaxis.

The previous results of PIONEER demonstrated that avapritinib significantly improved symptoms, reduced biomarkers of MC burden, and improved QoL, with a well-tolerated safety profile.^6^ Using the ISM-SAF, reductions in all individual symptoms were observed;^7^ however, this instrument is not designed to capture anaphylaxis. Also, anaphylactic reactions were not graded using an established system such as Ring and Messmer, which constitutes another limitation of the present study.^8^ Assessing the impact of treatment on the true rate of anaphylaxis and its severity is difficult based on the rarity of the events and the limited duration of both the non-interventional screening period used to determine baseline rates of anaphylaxis and the placebo-controlled interventional portion of the study. Based on this descriptive report of anaphylaxis events in patients receiving avapritinib versus placebo, larger studies with longer-term follow-up are warranted to fully characterize the effect of avapritinib treatment on anaphylaxis among patients with ISM.

## Abbreviations

BSC, best supportive care; ISM, indolent systemic mastocytosis; MC, mast cell; QoL, quality of life; SAF, symptom assessment form; SM, systemic mastocytosis; TSS, total symptom score.

## Data availability

All data generated or analyzed for this analysis are included in this article. Further enquiries can be directed to the corresponding author.

## Author contributions

All the authors have contributed substantially to the conception and design of the study, the acquisition, analysis, and interpretation of data. They all have contributed to the article drafting and have revised it critically for important intellectual content.

## Ethics

The trial was conducted according to the Declaration of Helsinki and Good Clinical Practice guidelines. The independent ethics committee or institutional review board of each participating trial center approved the protocol. Patients provided written informed consent. An independent data and safety monitoring committee monitored safety throughout the trial.

## Consent for publication

All authors have approved the final version of the manuscript and agreed to the submission.

## Disclosure of the use of generative AI and AI-assisted technologies

Nothing to disclose.

## Role of the funding source

Editorial support was supported by Blueprint Medicines Corporation, a wholly-owned subsidiary of Sanofi, according to Good Publication Practice guidelines (https://doi.org/10.7326/M22-1460). The sponsor was involved in the study design, collection, analysis, and interpretation of data, as well as data checking of information provided in the manuscript. However, ultimate responsibility for opinions, conclusions, data interpretation, and manuscript writing lies with the authors.

## Declaration of competing interest

**TP** has acted as a consultant for and has received research funding from Blueprint Medicines Corporation, a wholly-owned subsidiary of Sanofi. **MC** has no conflict of interests to disclose. **CA** has received research funding from Blueprint Medicines Corporation, a wholly-owned subsidiary of Sanofi, and Cogent and is a consultant for Blueprint Medicines Corporation, a wholly-owned subsidiary of Sanofi, Cogent, and Novartis. **ID** has received advisory board fees from Blueprint Medicines Corporation, a wholly-owned subsidiary of Sanofi. **JG** is a consultant for Blueprint Medicines Corporation, a wholly-owned subsidiary of Sanofi, Cogent, Imago, Incyte, Kartos, Novartis, PharmaEssentia, and Protagonist Therapeutics, and has received grants from Novartis and Protagonist Therapeutics. **JPP** is a consultant, speaker, and/or has worked on scientific advisory boards for Alexion, Apellis, AstraZeneca, Blueprint Medicines Corporation, a wholly-owned subsidiary of Sanofi, Boehringer Ingelheim, Bristol Myers Squibb, Chugai Pharmaceutical, F. Hoffmann-La Roche, Novartis, Pfizer, and Sanofi-Pasteur. **IA-T** is a consultant/speaker for and has received honoraria from Blueprint Medicines Corporation, a wholly-owned subsidiary of Sanofi and Novartis. **CMC** has no conflict of interests to disclose. **SC** is a consultant/speaker for and/or has worked on advisory boards for AbbVie, Bristol Myers Squibb, GlaxoSmithKline, Jazz Pharmaceuticals, Novartis, Paladin Laboratories, and Pfizer. **P Vadas** has no conflict of interests to disclose. **VS** has no conflict of interests to disclose. **P Vachhani** has received advisory board fees from AbbVie, Amgen, Blueprint Medicines Corporation, a whollyowned subsidiary of Sanofi, Cogent Biosciences, CTI BioPharma Corp, Daiichi Sankyo, Genentech, GlaxoSmithKline, Incyte, MorphoSys, Novartis, Pfizer, Servier, and Stemline. **FW** has been a speaker for Blueprint Medicines Corporation, a wholly-owned subsidiary of Sanofi. **CAY** has received advisory board fees from Blueprint Medicines Corporation, a wholly-owned subsidiary of Sanofi and Cogent Therapeutics LLC. **IB** is a current employee of Blueprint Medicines Corporation, a wholly-owned subsidiary of Sanofi. **KN** is a current employee of Blueprint Medicines Corporation, a wholly-owned subsidiary of Sanofi. **DS** is a current employee of Blueprint Medicines Corporation, a wholly-owned subsidiary of Sanofi. **KH** is/was a consultant/speaker for ALK-Abelló, Allergopharma, Almirall, BioCryst, Blueprint Medicines Corporation, a wholly-owned subsidiary of Sanofi, Cogent, Galderma, KalVista, Leo, Menarini, Novartis, Otsuka, Pfizer, Sanofi, Takeda, and Thermo Fisher.

## References

[1] Mesa R.A., Sullivan E.M., Dubinski D. (2022). Patient-reported outcomes among patients with systemic mastocytosis in routine clinical practice: results of the TouchStone SM patient survey. Cancer.

[2] Pardanani A. (2013). How I treat patients with indolent and smoldering mastocytosis (rare conditions but difficult to manage). Blood.

[3] Pardanani A. (2021). Systemic mastocytosis in adults: 2021 update on diagnosis, risk stratification and management. Am J Hematol.

[4] AYVAKIT® (November 2024). https://www.blueprintmedicines.com/wp-content/uploads/uspi/AYVAKIT.pdf.

[5] AYVAKYT® Summary of product characteristics. Blueprint Medicines (Netherlands) BV; https://www.ema.europa.eu/en/documents/product-information/ayvakyt-epar-product-information_en.pdf. Accessed 3 April 2025.

[6] Gotlib J., Castells M., Elberink HO. (2023). Avapritinib versus placebo in indolent systemic mastocytosis. NEJM Evid.

[7] Castells M., Akin C., Hartmann K. (2025). Continued symptom and quality of life improvement with favorable safety shown with long-term avapritinib in indolent systemic mastocytosis. J Allergy Clin Immunol Pract.

[8] Ring J., Messmer K. (1977). Incidence and severity of anaphylactoid reactions to colloid volume substitutes. Lancet.

