# Supplementary material for: Anaphylaxis events in the PIONEER study of avapritinib in indolent systemic mastocytosis
Source: World Allergy Organ J. 2026 Mar 14;19(4):101352. doi: 10.1016/j.waojou.2026.101352 (PMC12999330; doi:10.1016/j.waojou.2026.101352)
Supplement: Multimedia component 1 [file mmc1.docx]

**SUPPLEMENTARY MATERIAL**

**Supplemental Table 1.** Demographics, clinical characteristics, and treatment history of patients who experienced anaphylaxis during screening or treatment with avapritinib (N = 13)

| **Patients with anaphylaxis**  **events** | **Age (years)** | **Sex** | **Time from diagnosis to randomization (years)** | **Known allergens/triggers** | **B findings** | **Ongoing omalizumab** | **Prior cytoreductive therapy/TKI** | **# of BSC medications** |
| --- | --- | --- | --- | --- | --- | --- | --- | --- |
| **Avapritinib, n = 10/141 during screening and interventional period^a^** | | | | | | | | |
| 1 | 39 | F | 11 | Emotional stress, anxiety | No | Yes | Hydroxyurea | 6 |
| 2 | 35 | F | 12 | Intramuscular injection of Moderna bivalent COVID-19 vaccine | Hypercelluar BM^b^ | No | No | 4 |
| 3 | 61 | F | 4 | Unknown | No | No | No | 4 |
| 4 | 50 | F | 6 | Wasp sting | No | No | No | 5 |
| 5 | 48 | M | 10 | Possible mental and physical stress, oral phenoxymethylpenicillin, contrast dye | No | No | No | 5 |
| 6 | 54 | F | 4 | Fish, pork | No | No | No | 2 |
| 7 | 39 | F | 2 | Adhesives, oral amoxicillin/clavulanate, contrast dye, insect bite, strawberries, pineapple, tomatoes, perfume, essential oil diffuser, oral tramadol hydrochloride, wine, oral acetaminophen, oral aspirin, oral nizatidine, pineapple cake, McDonald’s chicken sandwich, mint, dust mites | No | Yes | Interferon alpha, midostaurin | 9 |
| 8 | 56 | M | 1 | Unknown | No | No | No | 3 |
| 9 | 43 | F | 11 | Mosquito bite, honey, tomato-based products, yeast-fermented sourdough bread, spicy food, morphine,^c^ oral ethylmorphine, oral tramadol, oral ampicillin | No | No | No | 4 |
| 10 | 39 | F | 10 | Bee and wasp stings, contrast agents, physical stress, friction, cold/heat, alcohol | No | No | Interferon alpha, dasatinib, cladribine (2CdA), midostaurin | 6 |
| **Placebo, n = 3/71** **during screening and interventional period^d^** | | | | | | | | |
| 11 | 34 | F | 1 | Scented candle | No | Yes | No | 6 |
| 12 | 66 | F | 15 | Gastroenteritis | MC in BM,  tryptase >200 ng/mL^e^ | Yes | No | 7 |
| 13 | 30 | F | 3 | Emotional stress, anxiety | No | Yes | No | 6 |
| BSC, best supportive care; MC, mast cell; MDS, myelodysplastic syndrome; MPN, myeloproliferative neoplasm; TKI, tyrosine kinase inhibitor; WHO, World Health Organization.  ^a^In the avapritinib group, 4/141 (2.8%) patients experienced anaphylaxis events during treatment.  ^b^Patient had hypercellular BM with loss of fat cells, discrete signs of dysmyelopoiesis without substantial cytopenias, or WHO criteria for an MDS or MPN.  ^c^Route of morphine administration unknown.  ^d^In the placebo group, 3/71 (4.2%) patients experienced anaphylaxis events while on placebo.  ^e^B findings represent MC infiltration in BM >30% by histology and baseline serum tryptase >200 ng/mL. | | | | | | | | |
